# Association between shift work and brain age gap: a neuroimaging study using MRI-based brain age prediction algorithms

**DOI:** 10.3389/fnagi.2025.1650497

**Published:** 2025-08-29

**Authors:** Youjin Kim, Joon Yul Choi, Evgeny Petrovskiy, Wanhyung Lee

**Affiliations:** ^1^Department of Preventive Medicine, College of Medicine, Chung-Ang University, Seoul, Republic of Korea; ^2^Department of Biomedical Engineering, Yonsei University, Wonju, Republic of Korea; ^3^International Tomography Center, Siberian Branch of the Russian Academy of Sciences, Novosibirsk, Russia

**Keywords:** shift work, brain age gap, brain aging, neuroimaging, MRI, brain

## Abstract

**Background:**

Shift work is increasingly common and associated with numerous adverse health effects. Although studies show that shift work affects brain structure and neurological stress, its direct impact on brain aging remains unclear. Therefore, this study aims to investigate the association between shift work and brain aging using the brain age gap (BAG)—a neuroimaging biomarker calculated by comparing predicted brain age derived from structural magnetic resonance imaging (MRI) scans to chronological age.

**Methods:**

Structural MRI data (T1-weighted and T2-weighted) were collected from 113 healthcare workers, including 33 shift workers and 80 fixed daytime workers. Brain age was estimated using seven validated machine learning models. BAG was calculated as the difference between predicted brain age and chronological age. Statistical analyses, including ANCOVA, adjusted for chronological age, sex, intracranial volume (ICV), education level, and occupational type.

**Results:**

The association between BAG and shift work duration was also evaluated. Model performance varied (maximum R^2^ = 0.79) and showed systematic age-related bias, typically underestimating brain age in older participants. Unadjusted analyses initially indicated lower BAG values in younger shift workers. However, after covariate adjustments, shift workers consistently exhibited significantly higher BAG values, suggesting accelerated brain aging. Two models retained statistical significance despite adjustment for potential confounders. Longer shift work duration correlated with a decreasing BAG trend, suggesting potential neuroadaptive changes or selective retention of resilient workers.

**Conclusion:**

These findings demonstrate that shift work is associated with accelerated apparent brain aging, even after controlling for systematic model bias and demographic covariates. The observed reduction in BAG with extended shift work exposure may reflect adaptive or selective effects, emphasizing the need for longitudinal studies to clarify these mechanisms. This research highlights the importance of incorporating occupational exposures in neuroimaging and brain health investigations.

## 1 Introduction

The proportion of the elderly population has increased globally and is expected to accelerate in the future. In 2020, the number of individuals aged 60 or older reached approximately 1 billion, more than 2.5 times the number in 1980, and is projected to be 2.1 billion by 2050, according to the World Health Organization (WHO; World Health Organization [WHO], 2021a). This demographic transition is accompanied by a substantial increase in the prevalence of age-related diseases, particularly dementia. The number of individuals living with dementia is expected to increase 2.7-fold between 2021 and 2050, and the global economic burden is estimated to reach 2.8 trillion United States dollars (USD) by 2030 (World Health Organization [WHO], 2021b). These trends underscore the growing need to better understand and address the aging process and neurodegenerative changes at an early stage.

An emerging method in studying aging is the estimation of brain age using neuroimaging. This technique involves data-driven models based on machine learning and deep learning algorithms, trained on magnetic resonance imaging (MRI) data from healthy individuals to predict chronological age (Tanveer et al., 2023). The discrepancy between brain age and chronological age, known as the brain age gap (BAG), is associated with an increased risk of Alzheimer’s disease and reduced cognitive function (Millar et al., 2023; Stefaniak et al., 2024). Recent studies have explored factors associated with BAG, revealing potential links to various diseases, such as diabetes, schizophrenia, and HIV, as well as psychosocial factors, inflammatory markers, and hearing loss, all of which are known contributors to neurodegeneration (Cole et al., 2017; Chen et al., 2022; Jawinski et al., 2022; Nemati et al., 2024; Gu et al., 2025; Lee et al., 2025). Thus, BAG is now regarded as a biomarker of brain aging, and ongoing research could identify possible protective and detrimental factors influencing brain health (Cole and Franke, 2017; Franke and Gaser, 2019).

Shift work, including night shifts, is prevalent across various sectors, such as healthcare, manufacturing, and transportation (Jang, 2023). These work schedules are associated with adverse health outcomes, including circadian rhythm disruption, sleep disturbances, cardiovascular disease, metabolic disorders, and increased stress levels (Stufano et al., 2024; Ryu et al., 2025). Recent studies have extended these concerns to neurological health, indicating that irregular work hours may negatively affect brain structure and cognitive function (Choi et al., 2025; de Souza et al., 2025). For instance, shift work is linked to altered gray matter volume, cognitive performance decline, and an increased risk of dementia (Choi et al., 2025). Regarding brain senescence, the brain age index—a metric derived from sleep electroencephalography—is higher in night-shift nurses than in daytime workers (Yook et al., 2024). Although some controversy remains, a study reports that shift work increases dementia risk (Leso et al., 2021). However, most existing studies have focused on isolated aspects of brain function or structure, limiting understanding of the broader effects of shift work on brain health. This highlights the need for more comprehensive and sensitive biomarkers to investigate the overall influence of shift work on brain health.

Therefore, this study aims to investigate the association between shift work and BAG using a cross-sectional design with data from healthcare workers. We also examined whether BAG varies with the duration of shift work to explore potential long-term effects. To strengthen our findings, BAG was estimated using multiple brain age prediction models. The findings may offer valuable insights for understanding and mitigating the influence of shift work on brain health.

## 2 Materials and methods

### 2.1 Dataset and participants

This study was conducted using data from healthcare workers at a regionally representative tertiary hospital in 2023 (Lee et al., 2022). These datasets have been utilized in a published study (Jang et al., 2025). Of the 122 MRI scans collected, 113 were included in the final analysis after excluding participants with suboptimal MRI image quality or repeated measurements (*n* = 3) and those with missing data (*n* = 6). Baseline demographic variables included age, sex, and highest education level. Handedness was also recorded to aid accurate neuroimaging interpretation. Work-related data comprised employment duration and occupational roles such as physician, nurse, or other healthcare professions.

### 2.2 MRI data acquisition

Magnetic resonance imaging images were obtained using high-resolution structural imaging sequences. The structural scans included a 3D T1-weighted magnetization-prepared rapid gradient echo and 3D T2-weighted Sampling Perfection with Application-optimized Contrasts using different flip angle Evolution. Acquisition parameters for the T1-weighted magnetization-prepared rapid gradient echo sequence included repetition time = 1970 ms, echo time = 2.84 ms, inversion time = 991 ms, flip angle = 9 °, voxel size = 0.5 mm × 0.5 mm × 1 mm, with 192 slices, and scan time of 4 min 34 s. Parameters for the T2-weighted Sampling Perfection with Application-optimized Contrasts using different flip angle Evolution sequences included repetition time = 3200 ms, echo time = 509 ms, flip angle = 120 °, voxel dimensions of 0.5 mm × 0.5 mm × 1 mm, with a total of 192 slices, and a scan time of 7 min 1 s.

### 2.3 Brain age models

In this study, we applied seven previously developed brain age prediction algorithms to the dataset, which included the following: “brainageR,” a Gaussian process regression model by Hobday et al. (2022); “XGBoost,” a gradient boosting model from Kaufmann et al. (2019); “ENIGMA,” a ridge regression model developed by the ENIGMA consortium (Han et al., 2021); “pyment,” a fully convolutional network by Leonardsen et al. (2022); “DenseNet,” a DenseNet-121 based model from the MIDI consortium (Wood et al., 2022, 2024); “TSAN,” a two-stage convolutional neural network (CNN) model by Cheng et al. (2021); and “SynthBA,” a 3D U-Net with regression head by Puglisi et al. (2024). We selected widely used and open-access models, representing various modeling strategies. The computational environment was based on Ubuntu 22.04.5 LTS. Due to hardware limitations, TSAN was executed on Google Colaboratory. For models requiring preprocessing, we used FreeSurfer 7.4 (Fischl, 2012) and FSL 6.0 (Jenkinson et al., 2012). T1-weighted magnetization-prepared rapid gradient echo images were used for all models except DenseNet, which required T2-weighted Sampling Perfection with Application-optimized Contrasts using different flip angle Evolution images. Table 1 presents details for each model.

**TABLE 1:** Summary of brain age prediction models.

Alias	Approach	Model type	Input modality	Input data	Training sample size	Relevant code
brainageR	Machine learning	Gaussian process	T1w	Voxel wise tissue probability features	3,377	https://github.com/james-cole/brainageR
XGBoost	Machine learning	Gradient boost	T1w	Morphometric features	35,474	https://github.com/tobias-kaufmann/brainage
ENIGMA	Machine learning	Ridge regression	T1w	Morphometric features	2,188	https://photon-ai.com/enigma_brainage
pyment	Deep learning	Fully convolutional network	T1w	Spatial pattern	34,285	https://github.com/estenhl/pyment-public
DenseNet	Deep learning	DenseNet CNN	T2w	Spatial pattern	18,890	https://github.com/MIDIconsortium/BrainAge
TSAN	Deep learning	Two-stage CNN	T1w	Spatial pattern	4,610	https://github.com/Milan-BUAA/TSAN-brain-age-estimation
SynthBA	Deep learning	3D U-Net with regressor head	T1w	Spatial pattern	3,489	https://github.com/LemuelPuglisi/SynthBA

T1w, T1-weighted image; T2w, T2-weighted image; CNN, convolutional neural network.

### 2.4 Shift work and covariates

Shift work status was determined based on self-reported work schedules utilizing standardized questions adapted from the Korean Working Condition Survey and previous literature (Cho, 2023; Choi et al., 2025). Any work schedule that deviated from regular daytime hours (approximately 09:00–18:00 in Korea, including a 1-hour lunch break) was categorized as shift work. In our study, participants were healthcare workers employed in a tertiary hospital clearly classified into shift workers and non-shift (fixed daytime) workers. Shift workers were predominantly engaged in three-shift rotations (day, evening, and night shifts). Participants also reported the initiation date of their shift work, enabling precise computation of shift work duration. Participants’ occupations were categorized based on professional roles within the hospital into three distinct groups: (1) physicians (including attending physicians, residents, and medical specialists), (2) nurses (registered nurses primarily engaged in clinical patient care), and (3) other healthcare workers (medical technicians, administrative staff, radiology technologists, and laboratory personnel). Occupation was included as a categorical covariate in the statistical analyses. Education level was categorized into three groups based on the highest degree attained by participants: (1) high school graduate, (2) university graduate (bachelor’s degree), and (3) graduate school or higher (master’s degree, doctoral degree, or equivalent). Education level was treated as a categorical covariate in statistical analyses.

Additional covariates were included to control for potential confounders. Since machine learning and deep learning models tend to regress toward the mean, a *post hoc* bias correction using values derived from training or validation dataset is generally recommended (de Lange and Cole, 2020). However, since we used public pretrained models and had limited sample size, we could not fit the correction model. The primary aim of our study was not estimating absolute brain age but to compare BAG between groups, thus we adjusted for chronological age as a covariate when analyzing to avoid age-related bias. Sex and intracranial volume (ICV), calculated using FreeSurfer, were included as general covariates. To account for individual differences in cognitive reserve, either education level or occupation was considered. Given the limited heterogeneity in education level within our cohort, occupation was included to better capture this variability.

### 2.5 Statistical analysis

To evaluate the performance and applicability of the brain age prediction models to our datasets, we calculated the mean absolute error (MAE), root mean squared error (RMSE), coefficient of determination (R^2^), bias, and Spearman’s rank correlation coefficient (ρ). The MAE and RMSE reflect the prediction error in age estimates, while R^2^ and ρ represent the consistency between predicted and chronological age. Bias indicates systematic over- or underestimation (Chicco et al., 2021).

We calculated the BAG as the difference between predicted brain age and chronological age. Chronological age was computed by dividing the number of days between the MRI scan date and date of birth by 365.25.

To compare BAG according to shift work status, *t*-tests and analysis of covariance (ANCOVA) were performed separately for each prediction model. Chronological age, sex, ICV, and either education level or occupation type were included as covariates in ANCOVA. Among shift workers, trends in BAG were examined using locally weighted scatterplot smoothing regression based on the duration of shift work. Linear regression analyses were additionally conducted for each brain age prediction model between BAG and shift work duration.

Statistical analysis was conducted using R version 4.4.3 (R Foundation for Statistical Computing, Vienna, Austria) to evaluate model performance and visualize BAG trends by years of shift work. SAS version 9.4 (SAS Institute, Cary, NC, USA) was used for group comparisons.

### 2.6 Ethics statement

The study was performed in accordance with the ethical standards of the Declaration of Helsinki (1964) and its subsequent amendments. All procedures involving human participants were reviewed and approved by the Institutional Review Board of Chung-Ang University (IRB number: 1041078-20231024-HR-287). Written informed consent was obtained from all participants.

## 3 Results

### 3.1 Evaluation metrics for each model

**FIGURE 1:**
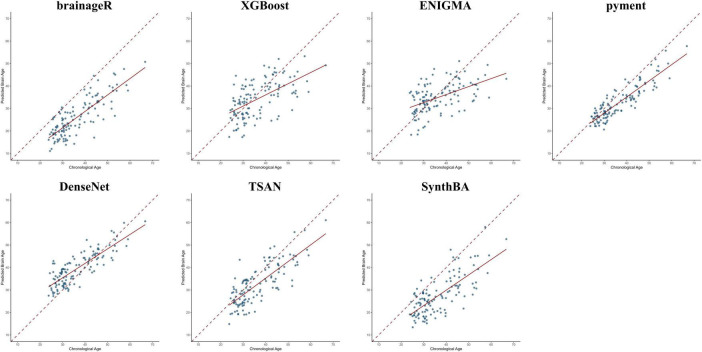
Scatter plots showing the relationship between chronological age and predicted brain age across all models.

**FIGURE 2:**
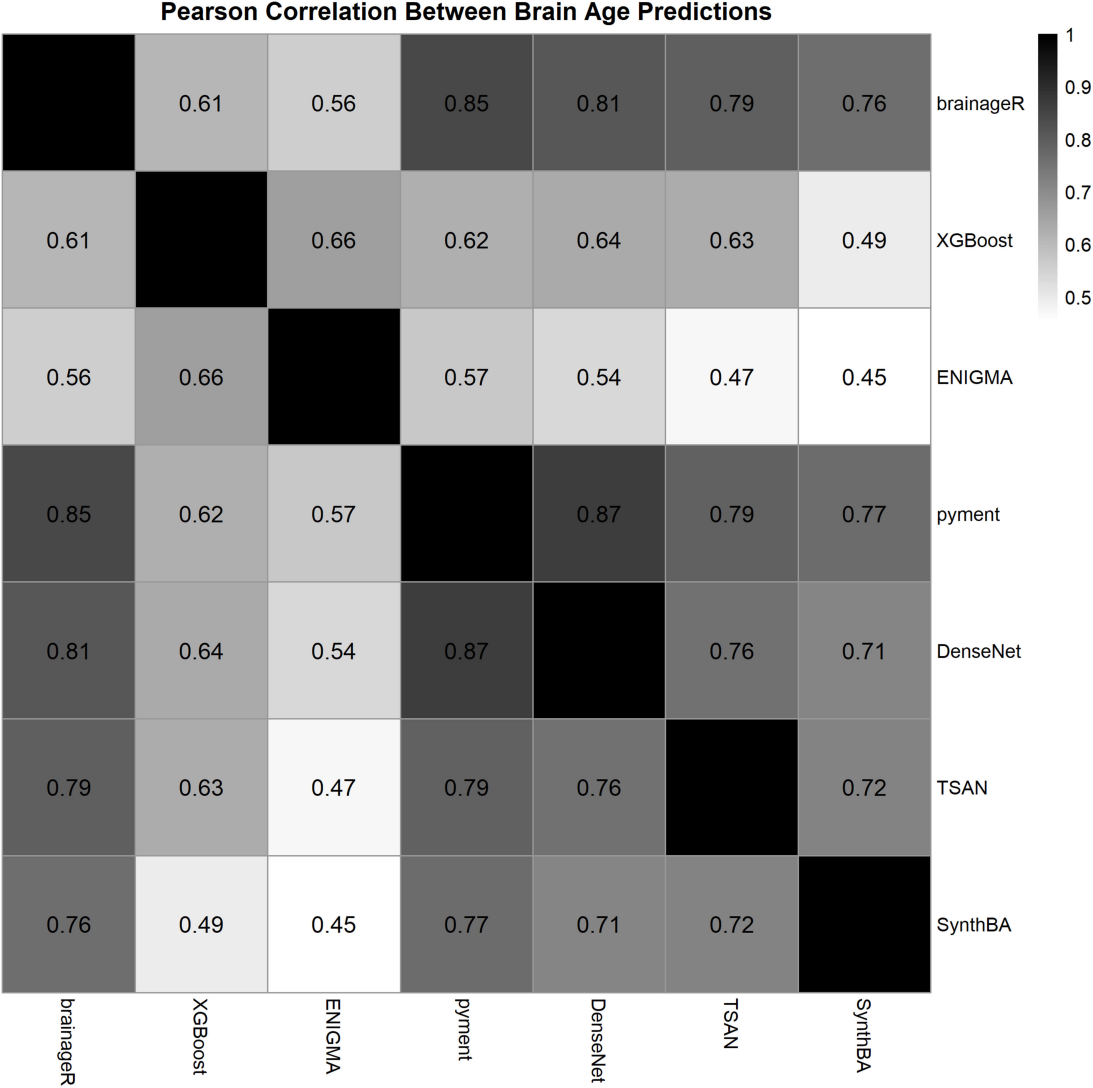
Pearson correlation coefficients between predicted brain ages from different models.

Table 2 presents the performance metrics of the seven brain age prediction models assessed on our dataset. Figure 1 displays scatter plots illustrating the relationship between chronological age and predicted brain age, with linear regression fits and identity lines (y = x) included as references. We primarily focused on model fit (R^2^ and ρ) rather than bias, as the main goal was to compare the BAG across shift work status. Among the models, pyment (R^2^ = 0.79, ρ = 0.85), DenseNet (R^2^ = 0.69, ρ = 0.78), brainageR (R^2^ = 0.66, ρ = 0.75), and TSAN (R^2^ = 0.61, ρ = 0.75) demonstrated strong explanatory power and high correlation with chronological age. SynthBA (R^2^ = 0.55, ρ = 0.68) exhibited moderate explanatory performance and relatively high correlation. In contrast, XGBoost (R^2^ = 0.33, ρ = 0.57) and ENIGMA (R^2^ = 0.27, ρ = 0.52) showed lower explanatory capabilities and moderate correlations. Figure 2 shows the Pearson correlations between the models; all *p*-values were < 0.001.

**TABLE 2:** Performance metrics of brain age prediction models.

Alias	R2	ρ	MAE	RMSE	Bias
brainageR	0.66	0.75	10.37	11.72	−10.37
XGBoost	0.33	0.57	6.66	8.39	−2.16
ENIGMA	0.27	0.52	6.38	8.34	−1.52
pyment	0.79	0.85	4.75	5.8	−3.87
DenseNet	0.69	0.78	4.92	6.08	3.12
TSAN	0.61	0.75	5.80	7.16	−3.87
SynthBA	0.55	0.68	9.38	11.08	−8.96

R2, coefficient of determination; ρ, Spearman’s rank correlation coefficient; MAE, mean absolute error; RMSE, root mean squared error.

### 3.2 Demographic characteristics

Table 3 summarizes the characteristics of the participants. Among 113 participants, 33 (29.2%) were shift workers. The mean age of shift workers was significantly lower than that of non-shift workers (39.1 ± 9.4 vs. 30.7 ± 6.3, *p* < 0.0001). Overall, the number of females was higher (63.7%), and the proportion of shift workers did not differ by sex (*p* = 0.9909). Most participants held a university degree (62.8%), followed by graduate degrees (36.3%), with one participant having completed only high school. Education level did not significantly differ by shift work status (*p* = 0.1725). However, occupational distribution varied significantly across shift work status (*p* = 0.0136) as follows: nurses (40.8%), doctors (31.0%), and other healthcare workers (11.4%). No significant difference in ICV was observed across shift work status (*p* = 0.4936).

**TABLE 3:** Demographic characteristics of study participants according to shift work status.

Characteristics	Total	Shift work, *n* (%) or mean ± SD		*p*-value
		No	Yes	
Total participants	113	80 (70.8)	33 (29.2)	
Age (years)	36.7 ± 9.4	39.1 ± 9.4	30.7 ± 6.3	<0.0001
Sex				0.9909
Male	41 (36.3)	29 (70.7)	12 (29.3)	
Female	72 (63.7)	51 (70.8)	21 (29.2)	
Education				0.1725
High school	1 (0.9)	1 (100.0)	0 (0.0)	
University	71 (62.8)	46 (64.8)	25 (35.2)	
Graduate	41 (36.3)	33 (80.5)	8 (19.5)	
Job				0.0136
Physician	29 (25.6)	20 (69.0)	9 (31.0)	
Nurse	49 (43.4)	29 (59.2)	20 (40.8)	
Others	35 (31.0)	31 (88.6)	4 (11.4)	
ICV (× 10^3^mm^3^)	1483.8 ± 179.5	1476.3 ± 179.9	1501.9 ± 180.1	0.4936

SD: standard deviation, ICV: intracranial volume. *P*-values are obtained using chi-squared test for categorical variables and *t*-test for continuous variables.

### 3.3 Shift work and brain age gap

To examine the association between shift work and BAG, independent *t*-tests followed by ANCOVA, which accounted for covariates, were conducted across multiple models (Table 4). Across all models used to estimate BAG, shift workers showed significantly higher BAG than that of non-shift workers (*p* < 0.05). After adjusting for chronological age and sex, only BAG estimates from the pyment (*p* = 0.0420) and TSAN (*p* = 0.0331) models remained statistically significant. This trend persisted after further adjustment for ICV (pyment: *p* = 0.0386; TSAN: *p* = 0.0354), ICV and education level (pyment: *p* = 0.0486; TSAN: *p* = 0.0398), and ICV and occupation instead of education (pyment: *p* = 0.0247; TSAN: *p* = 0.0468).

**TABLE 4:** Comparison of BAGs across models according to shift work status.

Alias	Total participants (mean ± SD)	Shift work (mean ± SD)		Statistics (*p*-value)				
		No	Yes	*t*-test	Analysis 1	Analysis 2	Analysis 3	Analysis 4
brainageR	−10.8 ± 5.4	−11.8 ± 5.7	−8.2 ± 3.8	**0.0001**	0.1348	0.1419	0.1513	0.1245
XGBoost	−2.5 ± 8.2	−3.7 ± 8.7	0.4 ± 6.2	**0.0070**	0.8944	0.9182	0.9039	0.7473
ENIGMA	−1.6 ± 8.5	−2.8 ± 9.2	1.3 ± 5.4	**0.0043**	0.1480	0.1559	0.1703	0.3080
pyment	−4.0 ± 4.3	−5.1 ± 4.1	−1.4 ± 3.8	**<0.0001**	**0.0420**	**0.0386**	**0.0486**	**0.0247**
DenseNet	2.8 ± 5.1	1.6 ± 5.1	5.7 ± 4.0	**<0.0001**	0.1275	0.1251	0.1382	0.1788
TSAN	−4.3 ± 5.9	−5.5 ± 5.6	−1.3 ± 5.5	**0.0005**	**0.0331**	**0.0354**	**0.0398**	**0.0468**
SynthBA	−9.2 ± 6.5	−10.0 ± 7.1	−7.4 ± 4.6	**0.0245**	0.9025	0.8926	0.966	0.6483

BAG, brain age gap; SD: standard deviation. Adjusted for (Analysis 1) age and sex; (Analysis 2) age, sex, and intracranial volume (ICV); (Analysis 3) age, sex, ICV, and education; and (Analysis 4) age, sex, ICV, and job. Bold values indicate significant differences in predicted brain age gap between group.

**FIGURE 3:**
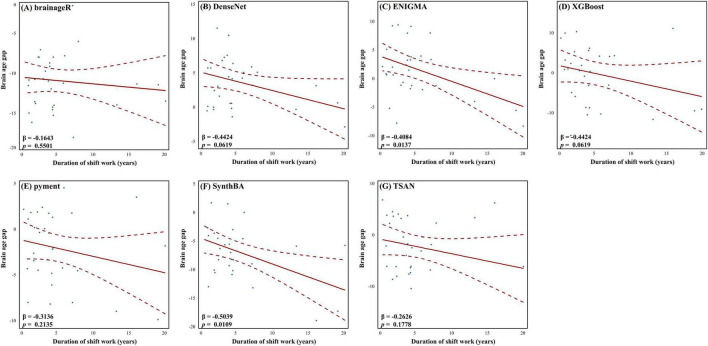
Relationship between duration of shift work and brain age gap (BAG) for each brain age prediction model. Solid lines represent estimated effects, and dashed lines indicate 95% confidence intervals. Linear regression results are included in each model with β and *p*-values.

We also visualized the relationship between the duration of shift work and BAG among shift workers (Figure 3). Throughout all brain age prediction models, BAG decreased with increasing shift work duration. Specifically, models such as ENIGMA (β = −0.4084, *p* = 0.0137) and SynthBA (β = −0.5039, *p* = 0.0109) demonstrated statistically significant linear associations, further reinforcing the presence of a negative relationship between shift work duration and BAG.

## 4 Discussion

In this study, we observed that shift workers exhibited significantly increased BAG compared to that of fixed daytime workers, indicating that irregular working schedules may accelerate brain aging. This association remained significant in certain brain age prediction models even after adjusting for relevant covariates, including chronological age, sex, ICV, education level, and occupational type. However, visual analyses of BAG trends among shift workers revealed a potential reduction in BAG with a longer duration of shift work. This trend may reflect adaptive neurological or selective survival of healthier individuals who continue long-term shift work. Longitudinal studies with larger samples are needed to further elucidate these dynamics.

Circadian disruption is a plausible biological mechanism underlying the association between shift work and accelerated brain aging observed in our study. Circadian rhythm disorders involve a chronic misalignment between the internal biological clock and external environmental signals of an individual (Meyer et al., 2022). They mainly occur in individuals with irregular or non-standard work schedules, such as shift workers (Boivin et al., 2022). This misalignment disrupts the normal synchronization of physiological processes with external time cues. The suprachiasmatic nucleus in the hypothalamus regulates endogenous circadian rhythms, controlling key functions, including the sleep–wake cycle, hormone secretion, metabolism, and cognitive processes (Patton and Hastings, 2018). Shift workers often experience irregularities in their circadian rhythm system, which negatively affects neural cell balance, neurogenesis, and synaptic plasticity (Wu et al., 2020; Leso et al., 2021). Circadian misalignment due to shift work can also increase oxidative stress and promote neuroinflammation, resulting in structural and functional alterations in the brain (de Souza et al., 2025). Over time, these disruptions may contribute to accelerated brain aging and heightened vulnerability to cognitive decline and neurodegenerative diseases.

The hypothalamic-pituitary-adrenal axis is a crucial neuroendocrine system involved in stress responses and the maintenance of physiological balance (Herman et al., 2020). Chronic stress, commonly experienced by shift workers due to irregular work schedules and sleep disorders (Liyanarachchi et al., 2017), activates this axis and leads to prolonged secretion of glucocorticoids, particularly cortisol. Sustained elevations in cortisol are well-documented to have harmful effects on brain health, especially on the hippocampus—a region essential for memory and learning (Tessner et al., 2007). Chronic cortisol elevation causes hippocampal atrophy, damages neuronal structural integrity, reduces synaptic density, and impairs neurogenesis (Echouffo-Tcheugui et al., 2018; Dronse et al., 2023). These structural and functional changes in the hippocampus result in cognitive decline and could accelerate overall brain aging (Dronse et al., 2023).

The observed decrease in the BAG among individuals with longer shift work durations suggests that chronic exposure to shift work may induce adaptive neural responses or compensatory mechanisms. These adaptations may involve long-term neuroplastic reorganization, similar to that documented in cognitively demanding professions, including musicians, pilots, and healthcare workers, where sustained environmental pressures lead to functional and structural brain changes (Wu et al., 2020). In shift workers, comparable mechanisms could help stabilize cognitive function and reduce BAG over time. This interpretation aligns with evidence showing that occupational physical stress is negatively associated with hippocampal volume and memory performance in older adults, indicating that work-related demands can influence brain structure in harmful and potentially adaptive ways depending on context and resilience (Burzynska et al., 2020). Additionally, long-term shift workers show attenuated cortisol responses compared to those of newly exposed workers, suggesting potential recalibration of stress-reactive systems (Boivin et al., 2022). While chronic circadian misalignment alters immune regulation, adaptive changes in microglial activity and B cell dynamics may help to limit neuroinflammation (de Souza et al., 2025). Altogether, these adjustments—across neural, endocrine, immune, and circadian systems—may contribute to the apparent resilience observed in long-term shift workers. However, not all individuals may benefit from such adaptations. Large-scale cohort studies still report elevated cognitive risk in night-shift populations, indicating that selective retention of more resilient individuals may be occurring (Khan et al., 2023). This phenomenon may reflect the “healthy worker effect” (Li and Sung, 1999), a form of selection bias. This effect suggests that individuals more resistant to the neurological stressors associated with shift work are more likely to remain employed, while more susceptible individuals tend to leave the workforce earlier. Longitudinal studies monitoring structural and functional brain changes over extended shift work durations are necessary to confirm the presence of true neurobiological adaptations and to distinguish them from selective survival effects.

We performed multiple analyses using various brain age prediction models, but only pyment and TSAN yielded statistically significant differences in BAG between shift workers and non-shift workers after adjusting for covariates. This inconsistency is likely due to differences in model architecture and input features. The machine learning models relied on structural brain features, while deep learning models are trained on raw spatial data (Table 1), potentially allowing them to capture more subtle and diffuse effects. Although the specific mechanisms remain unclear, this suggests that the impact of shift work on brain ageing may not be severe or localized, but rather mild and gradual, detectable only by more sensitive models. As interpretability remains a major limitation of deep learning, future advances in explainable artificial intelligence (XAI) are expected to provide further insight into these findings.

This study has some important strengths. A diverse array of brain age prediction models was employed, incorporating T1-weighted and T2-weighted MRI approaches.

Unlike previous studies that typically relied on a single model, this study presents several prediction methods, enabling cross-validation of BAG results. Furthermore, this analysis focuses on occupational characteristics (particularly shift work) that have been largely underexplored in neuroimaging studies, providing new insights into occupational neuroscience. The study also expands the applicability of brain age modeling by investigating brain aging processes in Asian populations, thus building on existing studies that primarily focus on Western populations. The findings were strengthened by adjusting for key confounding variables such as age, sex, ICV, education level, and occupational type. By conducting a dose-response analysis based on shift work duration, the study provides a more profound understanding of the potential adaptive or cumulative effects of long-term occupational exposure.

The findings of this study contribute to the advancement of neuroscience in the field of occupational health. The present study underscores the necessity of considering occupational factors when examining neurological aging by reporting the association between shift work and accelerated brain aging. Moreover, the observed neurological adaptations or potential selective survival effects among long-term shift workers suggest key directions for future research, including the need for longitudinal study designs. These findings also provide neuroscientific support for implementing occupational health strategies. Understanding the association between shift work and brain aging is essential for developing workplace interventions, including shift schedule management, rest during work hours, and sleep hygiene practices. Regular neurological health monitoring of shift workers may also be necessary.

This study has some limitations. First, its cross-sectional design precludes the establishment of a definitive causal relationship between shift work and brain aging. Longitudinal studies with repeated measures are required to clarify temporal and causal relationships. Second, although we utilized multiple brain age prediction models, their predictive accuracy varied among models, possibly due to differences in training datasets and imaging modalities. Most models were trained primarily on Western populations, potentially limiting their accuracy and generalizability in an Asian cohort. Therefore, developing brain age models specifically tailored to Asian populations may enhance predictive validity. Third, our sample was relatively homogeneous, consisting mainly of healthcare workers with high education levels, which may restrict the external validity and generalizability of the findings to other occupational groups or the broader population. Additionally, the observed trend of a decreasing BAG with longer durations of shift work may be confounded by the healthy worker effect, as individuals less resilient to shift work may have already exited the workforce. Lastly, unmeasured confounders, such as genetic predispositions, dietary habits, physical activity, detailed sleep patterns, and psychological factors, could influence the observed associations and were not fully accounted for in our analyses. Future research should incorporate comprehensive assessments of these factors to better elucidate the relationship between shift work and brain aging.

## 5 Conclusion

In conclusion, we observed that shift workers exhibited significantly higher BAGs than those of non-shift workers, indicating accelerated brain aging. Certain brain age prediction models retained statistical significance following adjustment for multiple confounders, including demographic and occupational variables. Additionally, BAG tended to decline with longer shift work durations, suggesting potential adaptive neurobiological responses or selection bias among long-term shift workers. These findings indicate that shift work negatively influences neurological health, highlighting the need for occupational health research and interventions aimed at reducing circadian disruption and mitigating neurological risk. Future longitudinal studies should explore the underlying biological mechanisms and the long-term implications of occupational shift work on cognitive health.

## Data Availability

The raw data supporting the conclusions of this article will be made available by the authors, without undue reservation.
